# Construction of recyclable homogeneous heterogeneous nanocones for enhanced photocatalytic uranium removal

**DOI:** 10.1039/d5ra06596h

**Published:** 2026-01-06

**Authors:** Xie Chen, Bohao Zhao, Jintao Wang, Yizhi Zeng, Yongtao Zhou, Guiming Chen, Feng Zhou, Yibing Guo

**Affiliations:** a High-Tech Institute of Xi'an Xi'an Shaanxi China 792757066@qq.com wangjintaolove@126.com zhouf_hgd@163.com; b The Third Geological and Mineral Exploration Institute of Gansu Provincial Bureau of Geology and Mineral Resources Lanzhou 730050 China 267255343@qq.com

## Abstract

The rapid expansion of nuclear power generation has played a crucial role in ensuring the sustainable utilization of global energy resources; however, it has concurrently intensified the risk of contamination from nuclear wastewater. Uranium, as the principal component of nuclear waste, poses significant environmental challenges. Photocatalytic adsorption has emerged as a promising method for the effective removal of dissolved uranium from aqueous solutions. Nonetheless, currently available photocatalytic materials predominantly exist in powder or coating forms, which limits their efficiency in rapidly capturing uranium within flowing nuclear wastewater. In response to this limitation, the present study reports the development of a novel catalytic architecture comprising homogeneous heterogeneous titanium dioxide (TiO_2_) nanocones coupled with multilayer carbon fibers. Specifically, the homogeneous heterojunction formed between anatase-phase TiO_2_ nanoparticles and rutile-phase nanocones facilitates efficient electron transport, while the multilayer support structure not only enhances recyclability but also improves the interfacial contact area for the reaction process. This catalyst demonstrates remarkable performance by removing approximately 92.97% of uranium from 100 mg L^−1^ uranium-containing wastewater within 10 minutes and exhibits rapid regeneration capability, maintaining over 90% uranium removal efficiency after 10 consecutive cycles. This work offers an effective strategy for uranium removal from wastewater, thereby contributing to the environmental sustainability of the nuclear energy industry.

## Abstract 2

The rapid expansion of nuclear power generation has played a crucial role in ensuring the sustainable utilization of global energy resources; however, it has concurrently intensified the risk of contamination from nuclear wastewater.

## Graphical abstract

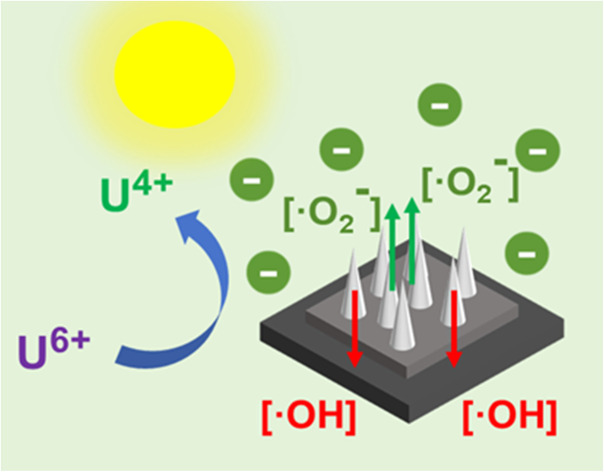

## 1. Introduction

Nuclear energy is considered a relatively ideal clean energy source and plays a critical role in ensuring the sustainable use of global energy resources.^1,2^ However, the nuclear power generation industry produces substantial volumes of uranium-containing radioactive wastewater throughout the uranium mining and nuclear fuel utilization processes, posing significant environmental risks.^3–5^ Due to the inherent radioactivity and the stable speciation of pollutants in nuclear wastewater, traditional biological metabolism^6,7^ and physical adsorption methods^8,9^ face considerable challenges in rapidly removing uranium from high-concentration nuclear effluents. In contrast, photocatalytic materials exhibit notable advantages such as structural stability under radiation exposure and the ability to transform pollutant species,^10,11^ enabling the efficient reduction of dissolved uranium in water to insoluble solid phases for rapid removal.^12–14^ Nonetheless, conventional photocatalysts are typically available as powders, which limits their long-term practical application in wastewater treatment.^15–17^ Therefore, recyclable approaches are necessary to ensure catalyst recovery and reuse.^18,19^

Current recyclable photocatalyst strategies predominantly rely on coating techniques.^20–22^ Researchers have fabricated recyclable photocatalytic disinfectants by directly adhering catalysts using adhesives,^23^ which facilitates material recycling and offers advantages of simple preparation and operation. Other studies utilize physical deposition methods,^24,25^ such as magnetron sputtering, to deposit catalyst coatings on various substrates, enabling photocatalytic treatment of multiple pollutants and providing adaptability to diverse substrate morphology. However, these methods—mainly involving physical or chemical approaches such as spraying or gluing catalyst powders onto recoverable substrates—face several limitations in uranium removal from nuclear wastewater.^26–28^ On one hand, coatings typically produce structurally uniform materials that lack complex architectures required for highly efficient catalysis, resulting in sub-optimal uranium removal performance.^29,30^ On the other hand, the substrates used in coating methods often possess dense, planar structures limiting catalyst loading capacity and reducing effective contact interfaces with uranium-containing wastewater.^31,32^

Hence, there is a compelling need to optimize recyclable photocatalyst design from materials structural perspective.^33–35^ To address these challenges, this study developed a recyclable uranium removal technology by integrating homogeneous heterogeneous nano-cone arrays with carbon fiber fabrics. Uranium extraction rate, capacity, and recyclability tests were conducted to evaluate how the nano-cone array structure enhances catalytic active surface area. Using electron microscopy and transient uranium adsorption experiments, the effect of hierarchical porous structures in carbon fibers on uranium separation efficiency was investigated. Additionally, through quenching experiments, electron paramagnetic resonance spectroscopy, photoluminescence spectroscopy, and band structure analyses, the mechanism by which the heterojunction interface formed between anatase-phase coatings and rutile-phase nano-cones promotes charge carrier separation was elucidated. This research advances photocatalytic uranium removal by synergistically improving charge carrier transport and materials structural design, thereby providing valuable insights for designing novel, highly efficient, and recyclable uranium separation materials.

## 2. Results and discussion

**Fig. 1:**
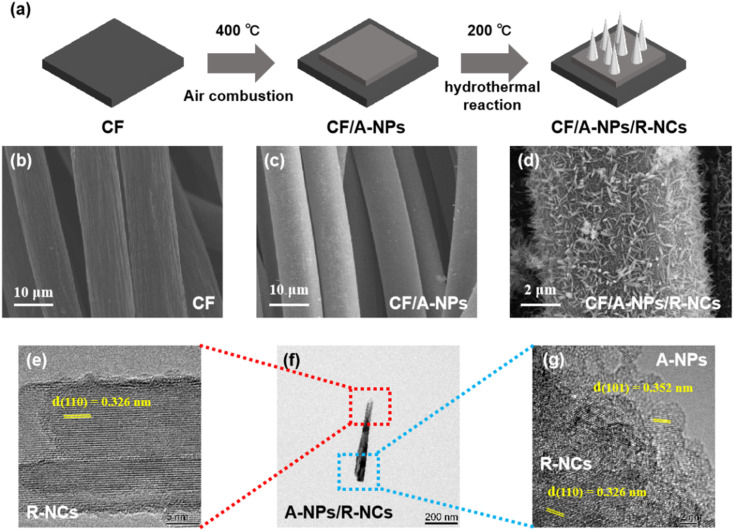
(a) Synthesis process of the samples. SEM images of the samples: (b) CF, (c) CF/A-NPs, (d) CF/A-NPs/R-NCs. TEM images of the samples: (e) R-NCs, (f) A-NPs/R-NCs, (g) A-NPs.

**Fig. 2:**
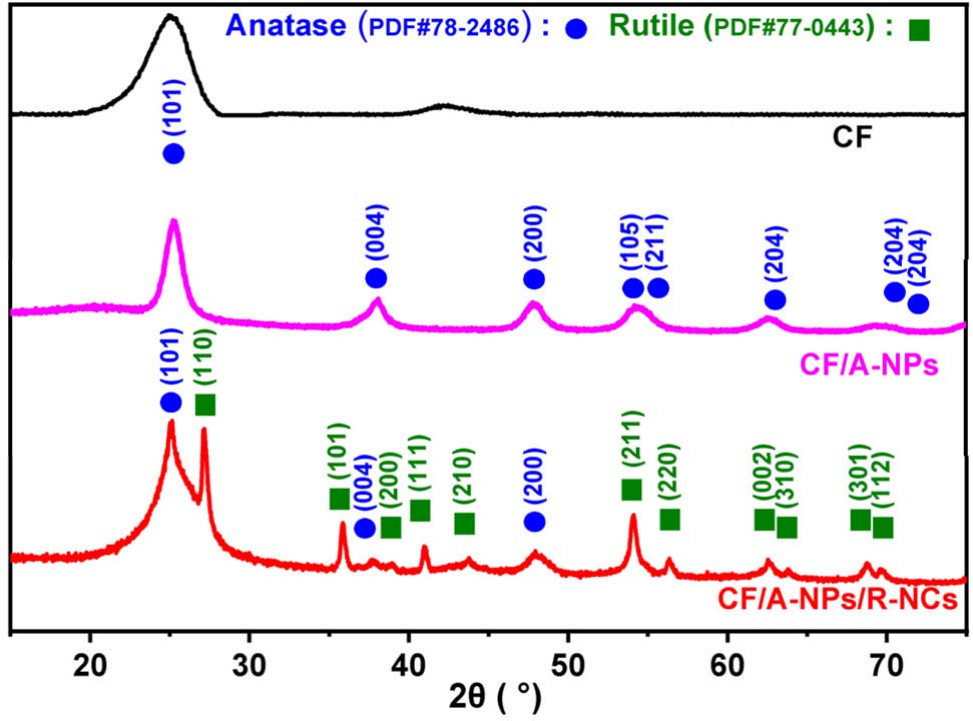
XRD patterns of the sample.

This study selected carbon fiber (CF) as the substrate material primarily because it possesses multiple advantageous properties, including excellent plasticity, strong resistance to corrosion, and a high capacity to bear mechanical loads.^36,37^ To fabricate the desired composite, the CF was first impregnated with a titanium precursor solution, after which it underwent a calcination process. This procedure yielded samples of carbon fiber that were uniformly loaded with anatase-phase titanium dioxide nanoparticles, referred to as CF/A-NPs. Following this, an additional step was carried out: rutile-phase titanium dioxide nanocones were grown directly and *in situ* on the surfaces of these pre-existing nanoparticles through a hydrothermal growth method, resulting in the final CF/A-NPs/R-NCs material. This material is characterized by a heterogeneous nanocone array structure, as illustrated in Fig. 1a. Morphological analysis of the entire sample series using scanning electron microscopy (SEM) revealed that the original CF consists of fibers approximately 10 µm in diameter (Fig. 1b), featuring a relatively rough surface that displays distinct vertical striations. In comparison, the CF/A-NPs sample maintains fibers of a similar diameter but exhibits a comparatively smoother surface texture (Fig. 1c), which strongly suggests that the anatase-phase nanoparticles were loaded uniformly onto the carbon fiber surface. On the surface of the CF/A-NPs/R-NCs sample, a dense distribution of nanocones is clearly visible (Fig. 1d); these fine and well-formed nanocones substantially increase the catalytic active surface area of the material, enhancing its functional properties. Further detailed examination using transmission electron microscopy (TEM) was performed on the active components. This analysis revealed that the anatase nanoparticles combined with the rutile nanocones (A-NPs/R-NCs) create nanocones measuring approximately 500 nm in length and ranging from 5 to 30 nm in width (Fig. 1f). More precisely, the lattice fringe spacing of the rutile-phase nanocones is measured to be 0.326 nm (Fig. 1e), corresponding well with the (110) crystal plane of rutile-phase titanium dioxide. Meanwhile, the lattice fringe spacing of the anatase-phase nanoparticles is 0.352 nm (Fig. 1g), which matches the (101) crystal plane of anatase-phase titanium dioxide. Additional characterization by X-ray diffraction (XRD) analysis showed that the CF/A-NPs sample, which contains only the anatase nanoparticles, presents a single-phase diffraction pattern (Fig. 2) corresponding to the standard reference card number 78-2486. This confirms that the nanoparticles attached to the carbon fiber surface are indeed of anatase phase. In contrast, the CF/A-NPs/R-NCs sample exhibits composite diffraction peaks that correspond to card numbers 78-2486 and 77-0443, indicating the presence of both anatase and rutile phases. The rutile phase corresponds to the nanocones grown on the anatase nanoparticle surface, thereby confirming the formation of the composite heterostructure, in agreement with the TEM results. In addition, this study analyzed the specific surface area of the materials through BET testing. The results showed that the pore volumes of CF/A-NPs and CF/A-NPs/R-NCs were 0.273 cm^3^ g^−1^ and 0.589 cm^3^ g^−1^ (Fig. S1), respectively, indicating that the conical structure effectively increased the active surface area of the materials, which is beneficial for the interfacial extraction of uranium. Overall, these comprehensive results demonstrate that, by employing the combination of impregnation-calcination and hydrothermal growth techniques, this study has successfully constructed a novel heterogeneous nanocone array directly on the surface of carbon fiber substrates.

**Fig. 3:**
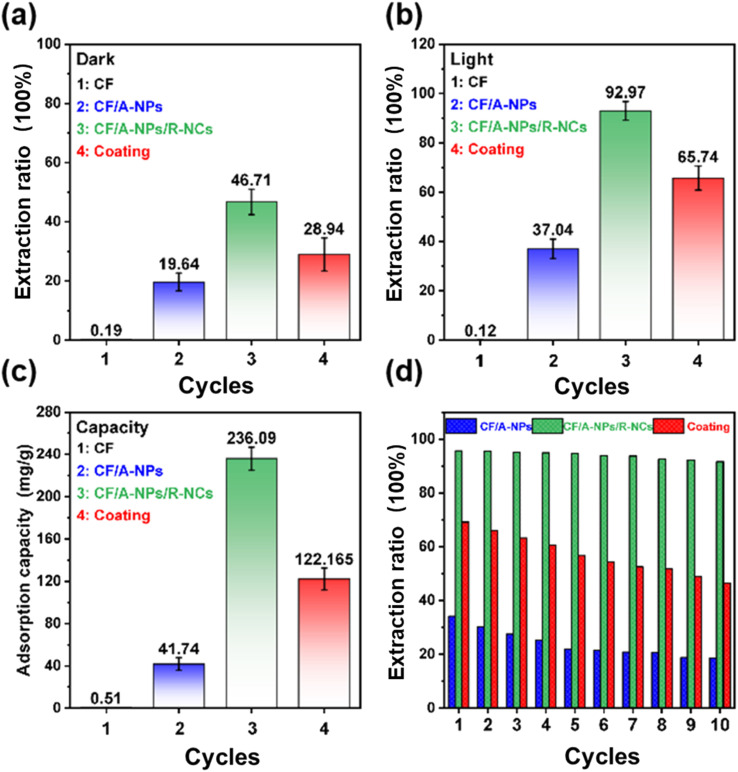
(a) Uranium adsorption effect in a dark environment, (b) photocatalytic uranium removal effect, (c) adsorption capacity of uranium removal material, (d) recycling performance of uranium removal material.

After thoroughly analyzing the morphological advantages of the materials under investigation, this study proceeded to evaluate their efficiency in removing uranium from aqueous solutions. Specifically, dark adsorption removal experiments were conducted using a simulated uranium-containing wastewater solution with an initial uranium concentration of 100 mg L^−1^. In these tests, pure carbon fibers demonstrated minimal adsorption capacity for uranium, indicating their limited ability to capture uranium ions in the absence of light. In contrast, the composites CF/A-NPs, CF/A-NPs/R-NCs, and coating exhibited noticeably improved uranium removal performances, achieving removal rates of 19.64%, 46.71%, and 28.94%, respectively, within just 10 minutes of contact time (Fig. 3a). These results clearly indicate that the titanium dioxide-based materials inherently possess a moderate uranium adsorption capability. Moreover, the combination of multi-layered carbon fibers with the distinctive nanoconical array structure found in CF/A-NPs/R-NCs significantly enhances the uranium adsorption effect beyond what is achievable by the simpler CF/A-NPs or coating samples. When the experiments were performed under illumination conditions, the uranium removal efficiencies improved substantially. Specifically, the removal efficiencies for CF/A-NPs, CF/A-NPs/R-NCs, and coating rose to 37.04%, 92.97%, and 65.74%, respectively (Fig. 3b). This notable enhancement under light exposure highlights the important role of photogenerated charge carriers in facilitating uranium removal. In particular, the CF/A-NPs/R-NCs sample showed a remarkable advantage, thanks to its optimized carrier structure and catalyst morphology, which together contribute to its superior photocatalytic uranium removal performance. This study also fitted the uranium removal amount based on time variation, with results following a linear trend (Fig. S2). The *k* values for CF/A-NPs/R-NCs, coating, and CF/A-NPs/R-NCs were 0.357, 0.526, and 0.741 (35.7 µg min^−1^, 52.6 µg min^−1^, 74.1 µg min^−1^), respectively, indicating that the structural advantage of the nanoneedles brings a significant benefit in extraction rate. To further quantify the uranium adsorption capacity of each material, this study measured the maximum uranium uptake, revealing that CF/A-NPs/R-NCs attained the highest adsorption capacity of 236.09 mg g^−1^ (Fig. 3c). This value was significantly greater than those observed for CF/A-NPs and coating samples, underlining the superior effectiveness of the heterostructured nanocone array in sequestering uranium. In addition, the cycling stability of the materials was assessed by subjecting them to repeated uranium removal tests over ten consecutive cycles. Impressively, CF/A-NPs/R-NCs retained approximately 90% of their initial uranium removal efficiency even after ten cycles of reuse (Fig. 3d). Which confirmed the reusability of CF/A-NP/R-NC removal efficiency and desorption rate of U(vi) and facilitated their practical applications (Table S1). By comparing with the literature, the advancement of this material is highlighted. The table includes the parameters you specified: photocatalyst name and concentration, initial uranium concentration, uranium VI to IV conversion percentage, reaction time, and light source with its intensity. In contrast, both CF/A-NPs and coating samples exhibited a gradual decline in uranium removal efficiency with each subsequent cycle, indicating poorer durability and reusability. To better elucidate the practical application potential of CF/A-NPs/R-NCs for photocatalytic uranium removal, these materials have demonstrated promising performance in laboratory-based uranium removal tests; however, their practical applicability in natural aquatic environments remains unclear.

Altogether, these findings clearly demonstrate that the presence of the nano-cone array structure plays a critical role in significantly enhancing the uranium separation performance of the composite materials, providing a promising approach for efficient and sustainable uranium removal from wastewater streams.

**Fig. 4:**
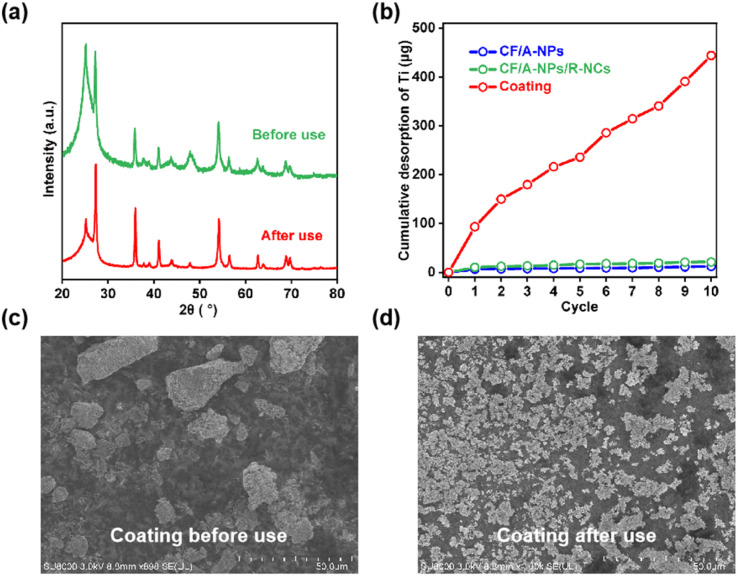
(a) Comparison of XRD patterns of CF/A-NPs/R-NCs before and after use, (b) titanium element shedding of the material during repeated use. SEM images of the coating sample (c) before and (d) after use.

To gain a deeper understanding of the factors contributing to the relatively high uranium removal efficiency exhibited by the CF/A-NPs/R-NCs material, this study first focused on analyzing its structural stability throughout the removal process. X-ray diffraction (XRD) testing was employed to examine any potential changes in the crystal structure of CF/A-NPs/R-NCs before and after uranium adsorption. The XRD results demonstrated that the crystal structure remained largely intact, showing no significant alterations after uranium removal (Fig. 4a). Notably, the diffraction peak intensity corresponding to the anatase phase around 25° was observed to be weaker compared to that of the rutile phase. This decrease in the anatase peak intensity could be attributed to the coverage of the TiO_2_ nanoparticles by adsorbed uranium species, which may partially obscure the diffraction signals. However, since the quantity of uranium adsorbed onto the material was below the detection threshold of the XRD instrument, this limited mass was not sufficient to cause noticeable distortion or changes in peak shapes or positions. The full XPS spectrum of CF/A-NP/R-NCs shows that the ratio of titanium atoms to oxygen atoms is basically 1 : 2 (Fig. S3), indicating that the catalytic structure is mainly titanium dioxide. Additionally, the uranium content reaches 0.41%, which indirectly confirms that the nanocone structure can capture a large amount of uranium from wastewater. To further assess the robustness of the material, the study also investigated the possible detachment or leaching of TiO_2_ catalysts from the composite structure during the uranium removal process. This was done by measuring the release of titanium ions into the solution as an indicator of catalyst stability. The testing results clearly showed that both CF/A-NPs/R-NCs and CF/A-NPs samples exhibited relatively stable behavior with minimal titanium release (Fig. 4b), suggesting that the catalysts remained securely anchored to the carbon fiber substrates. In contrast, the coating sample demonstrated a considerably higher rate of catalyst detachment, which provides a convincing explanation for its inferior uranium removal performance. To illustrate the physical changes occurring in the materials, SEM analyses were conducted on the coating sample before and after use. Prior to usage, the coating sample displayed uniformly distributed particles of varying sizes spread over the surface (Fig. 4c). However, after exposure to the uranium-containing solution under continuous flow conditions, nearly all of the larger particles, particularly those exceeding tens of micrometers in size, had disappeared from the surface (Fig. 4d). This observation reflects the inherent limitations and vulnerability of the coating structure when subjected to prolonged water flow erosion, a phenomenon that has also been reported in previous studies. Taken together, these findings underscore the superior structural integrity and catalyst retention of the CF/A-NPs/R-NCs composite, which are key factors underpinning its enhanced and stable uranium removal performance compared to other tested materials.

**Fig. 5:**
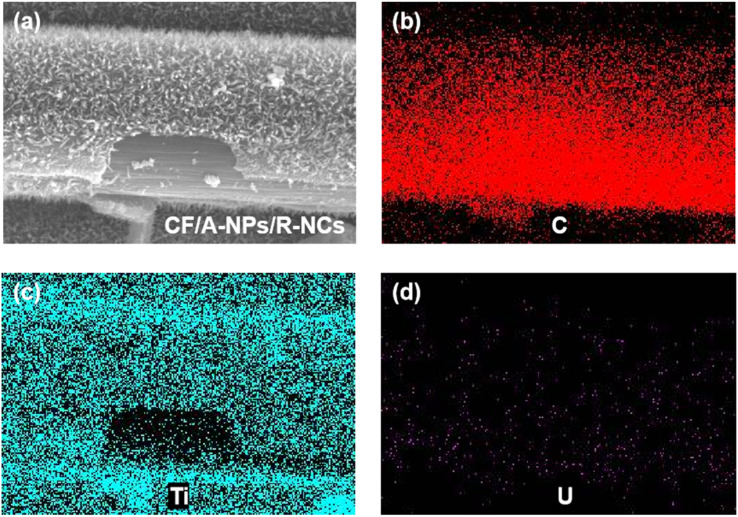
(a) SEM image of CF/A-NPs/R-NCs after uranium removal and elemental distribution on their surfaces: (b) carbon, (c) titanium, (d) uranium.

**Fig. 6:**
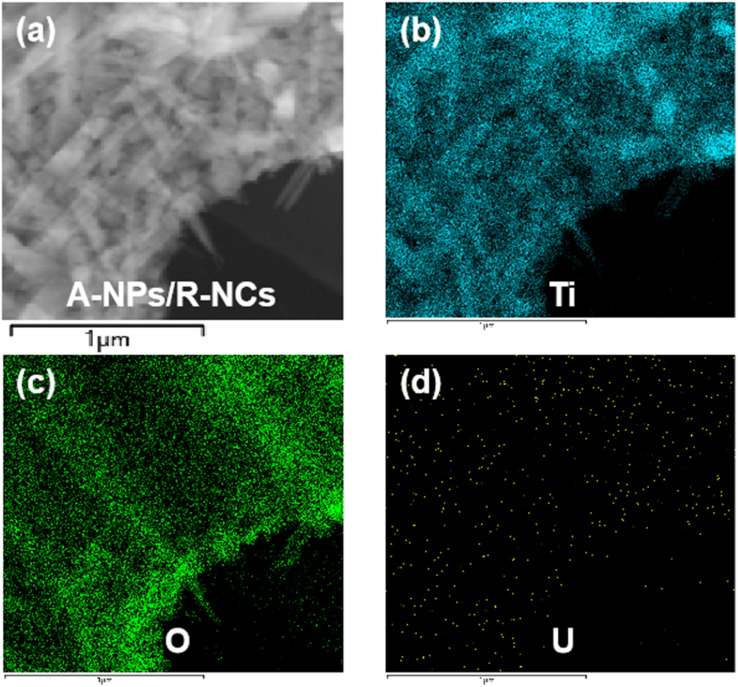
(a) TEM image of A-NPs/R-NCs after uranium removal and elemental distribution on their surfaces: (b) titanium, (c) oxygen, (d) uranium.

In comparison, the surface structure of the CF/A-NPs/R-NCs samples remained relatively intact and well-preserved even after undergoing the uranium removal process (Fig. 5a). Elemental mapping further confirmed this observation: both carbon (Fig. 5b) and titanium (Fig. 5c) elements exhibited a uniform distribution across the sample surface, with a substantial degree of spatial overlap. This strong correlation between carbon and titanium element distributions indicates that the anatase-phase nanoparticles and rutile-phase nanocones (A-NPs/R-NCs) remained firmly and stably attached to the underlying carbon fiber (CF) substrate. Importantly, this stable attachment enabled the composite material to effectively withstand continuous and rigorous flushing by simulated uranium-containing wastewater without significant loss or degradation of its structural features. Additionally, the uranium elemental distribution map showed a notable overlap with the titanium elements (Fig. 5d), which clearly demonstrates that the uranium ions removed from the aqueous solution were successfully adsorbed onto the surface of the material, specifically on the TiO_2_-based components. To gain further insight into the uranium adsorption behavior at a finer scale, TEM combined with elemental mapping was performed to analyze the adsorption of uranium by the A-NPs/R-NCs structure (Fig. 6a). The resulting data revealed a high degree of overlap between the distributions of titanium (Fig. 6b) and oxygen (Fig. 6c), allowing clear identification of the characteristic morphology of the TiO_2_ nanocones. Moreover, the uranium distribution (Fig. 6d) was found to coincide generally with the regions occupied by titanium and oxygen, which confirms that uranium adsorption occurs predominantly on these TiO_2_ nanostructures. However, the uranium spot density did not perfectly overlap with the titanium and oxygen signals, indicating that uranium atoms are adsorbed not only on the nanoparticles but also on the nanocones simultaneously. This spatial distribution suggests a synergistic adsorption mechanism involving both components of the composite material. Taken together, these results strongly indicate that both the anatase-phase nanoparticles and the rutile-phase nanocones within the CF/A-NPs/R-NCs composite actively participate in the adsorption and removal of uranium from solution. This dual participation highlights a significant structural and functional advantage of CF/A-NPs/R-NCs over materials that contain only a single active component such as CF/A-NPs or A-NPs/R-NCs coatings, which may lack such comprehensive adsorption capabilities and stability.

**Fig. 7:**
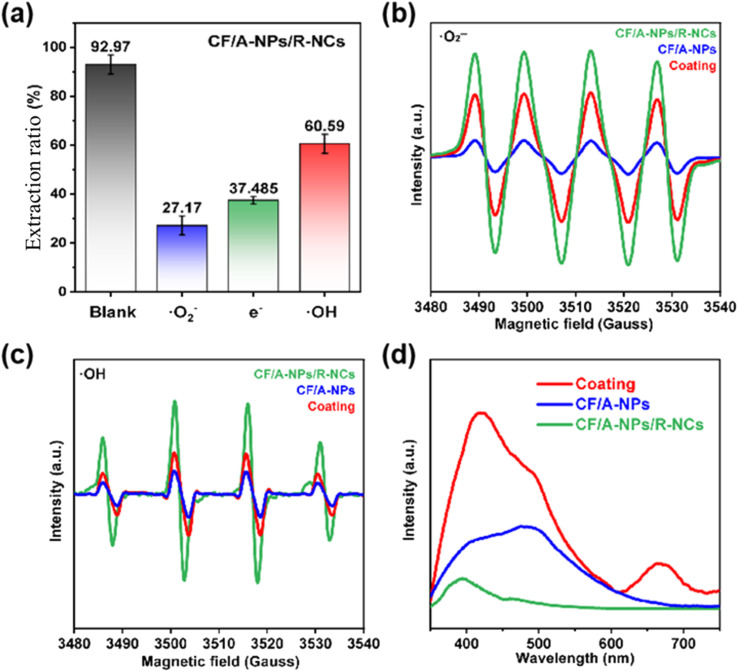
(a) Quenching experiment of active species in photocatalytic uranium removal by CF/A-NPs/R-NCs, (b) relative generation of superoxide radicals and (c) hydroxyl radicals under illumination. (d) Photoluminescence spectrum of the sample.

Furthermore, this study conducted a detailed investigation into the underlying reasons for the outstanding photocatalytic uranium removal performance exhibited by the CF/A-NPs/R-NCs composite, focusing particularly on the generation of active species during the photocatalytic process. To achieve this, specific chemical scavengers were employed to selectively quench distinct reactive species produced during photolysis. In the experiments, *p*-benzoquinone was used to quench superoxide radicals (˙O_2_^−^), ferric chloride served as an electron scavenger to capture photogenerated electrons (e^−^), and isopropyl alcohol was applied to quench hydroxyl radicals (˙OH). Following the addition of each respective quencher, the uranium removal efficiency of CF/A-NPs/R-NCs was measurably reduced, strongly indicating that the species targeted by these quenchers play critical and prominent roles in the photocatalytic uranium removal reaction. Based on the observed extent of efficiency reduction, the study determined the relative importance of the active species generated in this photocatalytic system, ranking them from strongest to weakest in terms of their contribution to uranium removal as follows: superoxide radicals, photogenerated electrons, and hydroxyl radicals (Fig. 7a). To further support these findings, EPR spectroscopy was performed, which provided direct evidence of the active species generated by CF/A-NPs/R-NCs under light illumination. The EPR results revealed a significant generation of superoxide radicals (Fig. 7b) as well as hydroxyl radicals (Fig. 7c), underscoring the material's robust capacity to produce reactive species that contribute to the efficient removal of uranium. Complementary to this, photoluminescence (PL) spectroscopy was used to assess the charge carrier dynamics within the CF/A-NPs/R-NCs structure. The PL spectra showed that CF/A-NPs/R-NCs exhibit noticeably weaker fluorescence emission under light excitation (Fig. 7d), which is indicative of more effective charge separation and reduced recombination of photogenerated electron–hole pairs within the catalyst. The large number of electron holes generated by carrier separation can react with oxygen and water in solution, producing superoxide radicals (Formula 1) and hydroxyl radicals (Formula 2), which act as potent active species to accelerate uranium extraction. Because of this superior charge separation efficiency, the material is able to generate a greater number of excited-state photogenerated electrons when irradiated with light of equivalent intensity compared to other catalyst systems. Therefore, the ability of CF/A-NPs/R-NCs to produce a substantial quantity of free radicals (especially superoxide and hydroxyl radicals) along with photogenerated electrons is identified as one of the key factors responsible for its highly efficient photocatalytic uranium removal capability. Furthermore, electrochemical impedance testing and photocurrent excitation testing also show that CF/A-NPs/R-NCs have lower impedance (Fig. S4a) and higher photocurrent response (Fig. S4b), which demonstrates that the formation of the nanocone structure comprehensively promotes the generation and transfer of photogenerated charges, thereby indirectly enhancing the apparent quantum efficiency. This enhanced generation of reactive species ensures that the composite can facilitate uranium adsorption and catalytically degrade uranium species more effectively than materials with less efficient charge separation and active species production.

1 O_2_ + e^−^ → ˙O_2_^−^

2 H_2_O + h^+^ → ˙OH + H^+^

**Fig. 8:**
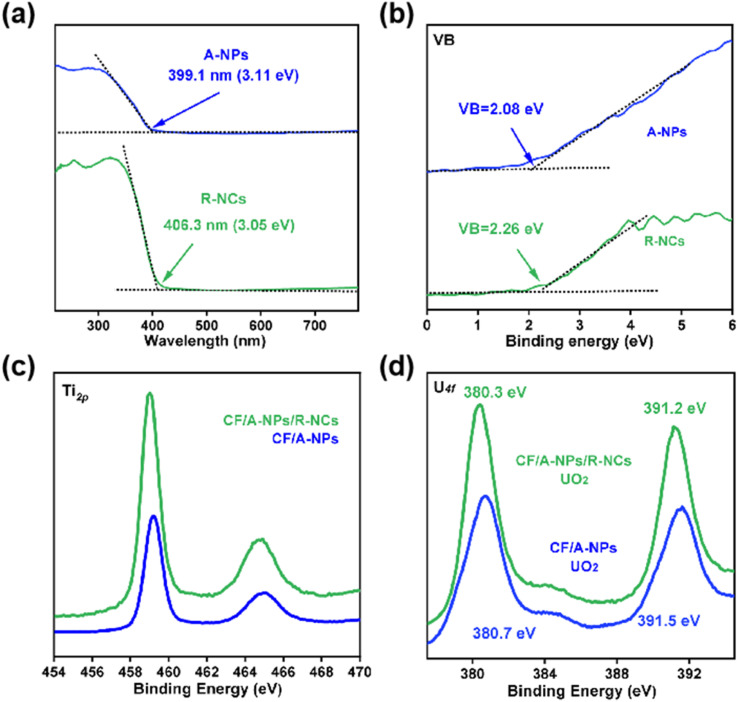
(a) UV-visible absorption spectra and (b) valence band spectra of A-NPs and R-NCs. (c) Titanium binding energy and (d) extracted uranium binding energy of the samples.

This study also conducted an in-depth discussion on the charge transfer mechanism occurring at the heterointerface by performing detailed band structure analysis, aiming to further elucidate the intrinsic factors responsible for the notably strong carrier separation effect and efficient generation of active species observed in the CF/A-NPs/R-NCs composite. Through analysis of the ultraviolet-visible absorption spectra, the bandgaps of the two involved semiconductor components—anatase-phase titanium dioxide nanoparticles (A-NPs) and rutile-phase nanocones (R-NCs)—were calculated to be 3.11 eV and 3.05 eV, respectively, as determined by extrapolating the intercepts of their absorption edges (Fig. 8a). Additionally, the valence band maximum energy levels for these two components were obtained from XPS valence band spectra, with the top positions identified as 2.08 eV for A-NPs and 2.26 eV for R-NCs (Fig. 8b). These band structure parameters imply that at the heterophase interface formed between the anatase and rutile phases, electrons energetically favor injection from the A-NPs into the R-NCs, while holes are simultaneously driven to migrate from the R-NCs back to the A-NPs. This bidirectional charge transfer establishes a type II heterojunction at the interface, which inherently promotes efficient spatial separation of photogenerated electron–hole pairs, thereby reducing recombination rates and enhancing photocatalytic activity. To experimentally verify this proposed charge transfer mechanism, the study further examined the binding energies of key elemental species within the CF/A-NPs and CF/A-NPs/R-NCs samples using XPS. The results revealed that CF/A-NPs/R-NCs exhibit a lower titanium element binding energy (Fig. 8c) relative to CF/A-NPs alone, indicating that the R-NCs component possesses a stronger electron affinity and thus acts as an effective electron sink. Moreover, the binding energy measurement of uranium dioxide species adsorbed on the surface of CF/A-NPs/R-NCs was found to be lower (Fig. 8d), suggesting that the presence of R-NCs enhances the interfacial reducibility of the composite. This enhancement is typically associated with improved electron donation capability, which further supports the conclusion that electrons preferentially transfer from A-NPs to R-NCs within the heterostructure. Collectively, these findings confirm that the heterointerface between the anatase nanoparticles and rutile nanocones facilitates improved charge transfer dynamics, leading to better photocatalytic performance. Based on existing literature, the binding energies of U(iv) are typically reported around 380.2 eV and 391.3 eV,^38^ while those of U(vi) are generally observed near 382.0 eV and 392.8 eV,^39^ demonstrating a significant differentiation in peak positions. The uranium peak positions observed in our study show strong alignment with the reported binding energies for U(iv) species. Since tetravalent uranium solid phases in environmental conditions are predominantly UO_2_, the product can be confidently identified as uranium dioxide.

**Fig. 9:**
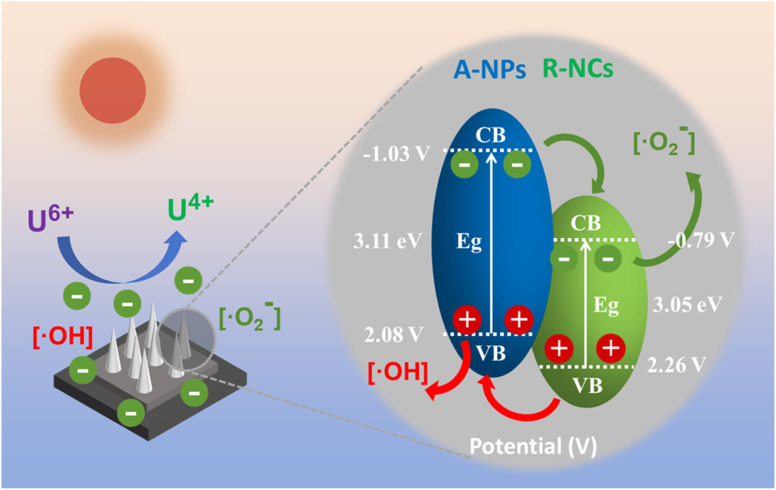
Schematic diagram of the mechanism of photocatalytic uranium removal by CF/A-NPs/R-NCs.

Building upon these insights, the photocatalytic uranium removal mechanism of the CF/A-NPs/R-NCs material, as developed in this study, is schematically illustrated in Fig. 9. Upon light irradiation, photogenerated electrons are injected from the anatase-phase nanoparticles into the rutile-phase nanocones, where they participate in the generation of superoxide radicals (˙O_2_^−^). Simultaneously, photogenerated holes migrate in the opposite direction from the rutile nanocones back to the anatase nanoparticles, resulting in the formation of hydroxyl radicals (˙OH). This effective bidirectional charge and radical generation process leads to an enrichment of photoexcited electrons and reactive radicals on the surface of the heterogeneous nanocone array. Consequently, these highly reactive species facilitate the efficient conversion of toxic hexavalent uranium (U(vi)) present in the solution into the less soluble and less toxic tetravalent uranium (U(iv)), thus achieving superior uranium removal performance.

## 3. Materials and methods

### 3.1. Chemical reagent

The carbon cloth used in this study was purchased from Carbon Energy Group, uranyl nitrate was obtained from Dingtian Chemical Group, and all other chemical reagents were sourced from Sinopharm Group.

### 3.2. Synthesis of photocatalytic materials

Square pieces of carbon fiber cloth measuring 3 cm × 3 cm were cut and immersed in an ethanol solution containing 1 vol% tetrabutyl titanate for 1 minute. The impregnated carbon cloth was then dried at 60 °C in an oven, followed by calcination at 400 °C for 2 hours in a muffle furnace to obtain carbon fiber-supported anatase-phase TiO_2_ nanoparticle-coated materials, denoted as CF/A-NPs.

Subsequently, 30 mL of 0.05 mol L^−1^ Na_2_EDTA aqueous solution, 3 mL of acetylacetone, and 0.5 mL of isopropyl titanate were sequentially added to a reaction vessel and stirred for 4 hours until the solution became clear. The CF/A-NPs were then introduced for a hydrothermal reaction at 200 °C for 15 hours. After completion, the products were washed with ethanol or water and dried to yield TiO_2_-based homogeneous heterogeneous nano-cone array materials, designated as CF/A-NPs/R-NCs.

Using tweezers to repeatedly squeeze CF/A-NP/R-NCs to the point of destroying the macroscopic structure, and further separating the A-NP/R-NCs structure from the CF carrier through strong ultrasound, the above operation was carried out in an ethanol solution containing 5% naphthol. Then, the suspension containing A-NP/R-NCs was continuously dropped onto carbon cloth of the same size, so that the ordered A-NP/R-NCs were transferred to a brand new carbon fiber, forming a coating material containing similar catalysts, which was named coating.

### 3.3. Photocatalytic uranium removal experiments

Recyclable catalysts with an exposed surface area of 9 cm^2^ were affixed to the inner wall of a beaker. Under simulated solar irradiation from a xenon lamp (intensity: 100 mW cm^−2^), 100 mL of uranyl nitrate solution (initial U concentration: 100 mg L^−1^) was added and stirred continuously. Samples were taken immediately before irradiation and after 10 minutes of reaction to measure uranium concentrations using inductively coupled plasma mass spectrometry (ICP-MS). Uranium removal efficiencies were calculated based on the concentration changes.

### 3.4. Characterization

The crystallographic morphology of the samples was thoroughly characterized using field emission transmission electron microscopy (FE-TEM), specifically employing a Tecnai G2F30 S-TWIN instrument from FEI, USA, operated at a high accelerating voltage of 300 kV to achieve high-resolution imaging. To complement this, detailed morphological images of the materials were acquired through scanning electron microscopy (SEM), utilizing a Hitachi SU8020 system from Japan, operated at an accelerating voltage of 5 kV, which provided clear surface topography and structural details. The crystalline phases present within the samples were systematically identified using X-ray diffraction (XRD) analysis, conducted on a Bruker D8 Advance diffractometer from Germany, facilitating precise phase determination and crystallinity assessment. To investigate the generation and presence of free radicals during photocatalytic processes, electron paramagnetic resonance spectroscopy (EPR) was employed, using a Bruker A300-10/12 model from Germany, which is highly sensitive to paramagnetic species and allowed for detection of reactive oxygen species. Elemental distribution and mapping across the samples were obtained *via* energy dispersive X-ray spectroscopy (EDS), performed on a HORIBA EMAX mics2 system from Japan operated at an accelerating voltage of 15 kV, enabling spatially resolved compositional analysis. Lastly, to analyze the valence band structures as well as detailed elemental compositions and chemical states present in the materials, X-ray photoelectron spectroscopy (XPS) was used, conducted on a Thermo ESCALAB 250Xi instrument from Thermo Fisher, USA, providing comprehensive surface chemical characterization.

## 4. Conclusion

In summary, this study successfully developed an advanced photocatalytic material for uranium removal that is based on carbon fiber substrates loaded with heterogeneous titanium dioxide nanocone arrays. This novel material design not only provides practical advantages such as convenient and efficient recovery of the material after use but also exhibits significantly enhanced resistance to water erosion compared to conventional powder coating-based catalysts, which tend to degrade or detach under flowing water conditions. The experimental results clearly demonstrate that the formation of the nanocone array structure on the carbon fiber surface leads to a substantial increase in the catalytic active surface area, which directly contributes to improved photocatalytic performance. Furthermore, the innovative combination of rutile-phase titanium dioxide nanoparticles with anatase-phase nanocones creates a synergistic heterojunction that greatly facilitates interfacial charge transfer. This enhanced charge separation reduces recombination losses and effectively boosts the production of reactive active species, such as superoxide and hydroxyl radicals, which are crucial for catalytic degradation and reduction of uranium species in solution. As a result, the photocatalytic uranium removal efficiency is significantly improved relative to single-phase or less structured catalysts. Overall, this work not only provides a promising new material platform for efficient and durable photocatalytic uranium removal but also serves as a valuable reference and foundation for future research and development in this field. Moreover, it expands the practical application potential of photocatalytic uranium remediation technologies, paving the way for more sustainable and scalable solutions to address uranium contamination issues in environmental water systems.

## Conflicts of interest

There are no conflicts to declare.

## Supplementary Material

RA-016-D5RA06596H-s001

## Data Availability

All experimental and characterization data supporting this article have been included in the supplementary information (SI) file. Supplementary information is available. See DOI: https://doi.org/10.1039/d5ra06596h.
