# Supplementary material for: Construction of recyclable homogeneous heterogeneous nanocones for enhanced photocatalytic uranium removal
Source: RSC Adv. 2026 Jan 6;16(2):1932–42. doi: 10.1039/d5ra06596h (PMC12772499; doi:10.1039/d5ra06596h)
Supplement: RA-016-D5RA06596H-s001 [file RA-016-D5RA06596H-s001.pdf]

Supplementary Information (SI) for RSC Advances.
This journal is © The Royal Society of Chemistry 2025

Construction of Recyclable Homogeneous Heterogeneous Nanocones Enhanced Photocatalytic Uranium Removal

Chen Xie [1], Bohao Zhao [1], Jintao Wang [1,*], Yizhi Zeng [1], Yongtao Zhou [1], Guiming Chen [1,*], Feng Zhou [1,*], and Yibing Guo [2,*]

[1] High-Tech Institute of Xi'an, Xi'an, Shaanxi, China
[2] The Third Geological and Mineral Exploration Institute of Gansu Provincial Bureau of Geology and Mineral Resources, Lanzhou 730050, China
* Correspondence: 792757066@qq.com (G.C.); 267255343@qq.com (Y.G.)

Table S1. Comparative Analysis of Uranium Extraction Performance: Capacity, Selectivity, and Reusability.

Material/System	Capacity	Selectivity	Reusability
TiO_2/CPAN-AO[1]	2.38 g/g (0.01 M uranyl solution, 5 h light)	High selectivity for UO_2^{2+} via amidoxime groups; ~95% reduction in ion-competitive seawater.	>99% efficiency after 10 cycles; no structural decay.
Mn-TiO_2@PO_4[2]	94% U(VI) removal (unspecified initial conc.)	Maintains >90% efficiency with K^+, Ca^{2+}, Na^+; 93% removal in fluoride-containing wastewater.	83% efficiency after 5 cycles; stable morphology.
TNTPAO[3]	30 μg/cm² (15 L salt lake, 96 h); 94.5% over 3 cycles	Selective via H-bonds/N atoms; resists Mg^{2+}, Li^+ in salt lakes.	93.6% recovery over 3 cycles; stable in real brine.
N_3-COF_{60} nanowires[4]	34.5 mg/g (42 days, commercial seawater)	Selective over Fe^{3+}, Co^{2+}, Ni^{2+}; exceeds commercial benchmark (30 mg/g) for seawater extraction.	No explicit cycles, but stable structure post-uptake.

PN-MOF10@5[5]	1590 mg/g (400 ppm uranyl, visible light)	Dual active sites (phosphonic acid/benzothiadiazole) enhance UO_2^{2+} coordination; verified by XPS/DFT.	817 mg/g capacity after 6 cycles (89% elution rate).
CMP-D-π-A[6]	11.68 mg/g (natural seawater, 42 days)	Light-enhanced selectivity (U:V ratio 3.11 vs. 1.87 in dark); antibacterial via $\cdot O_2^-$ g eneration.	89% elution rate over 6 cycles; no performance decay.
CF/A-NPs/R-NCs (In this study)	92.72% U(VI) removal (100mg/L)	High selectivity for UO_2^{2+}	Maintains high removal rate after 4 cycles.

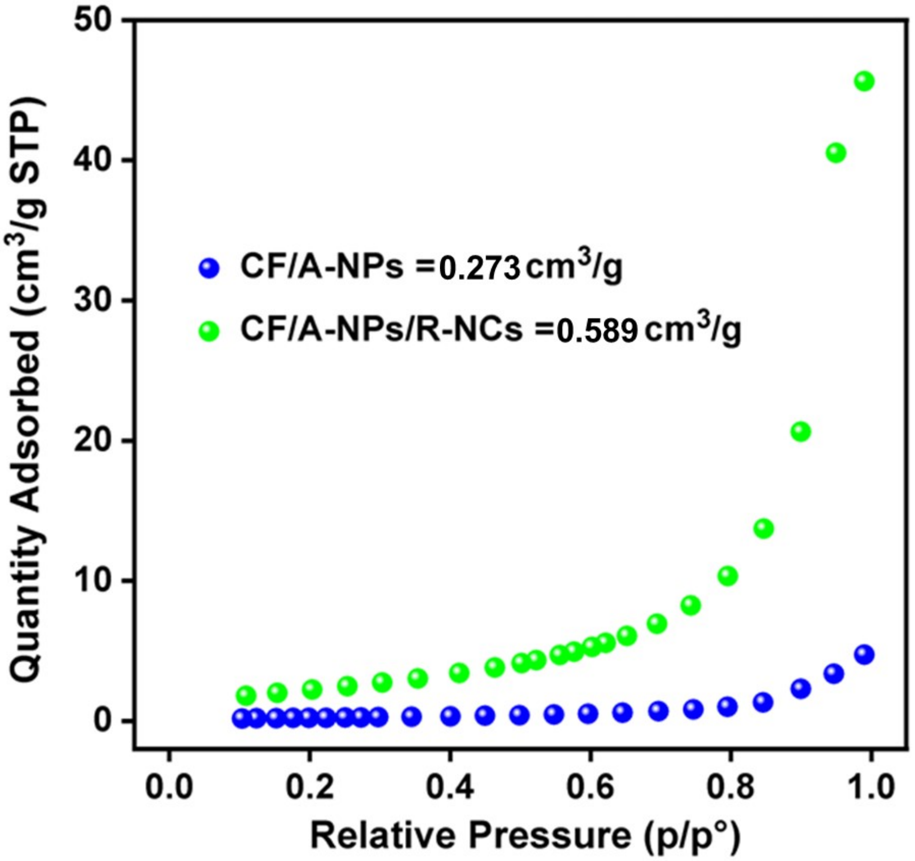

Figure S1. Specific surface area of the samples.

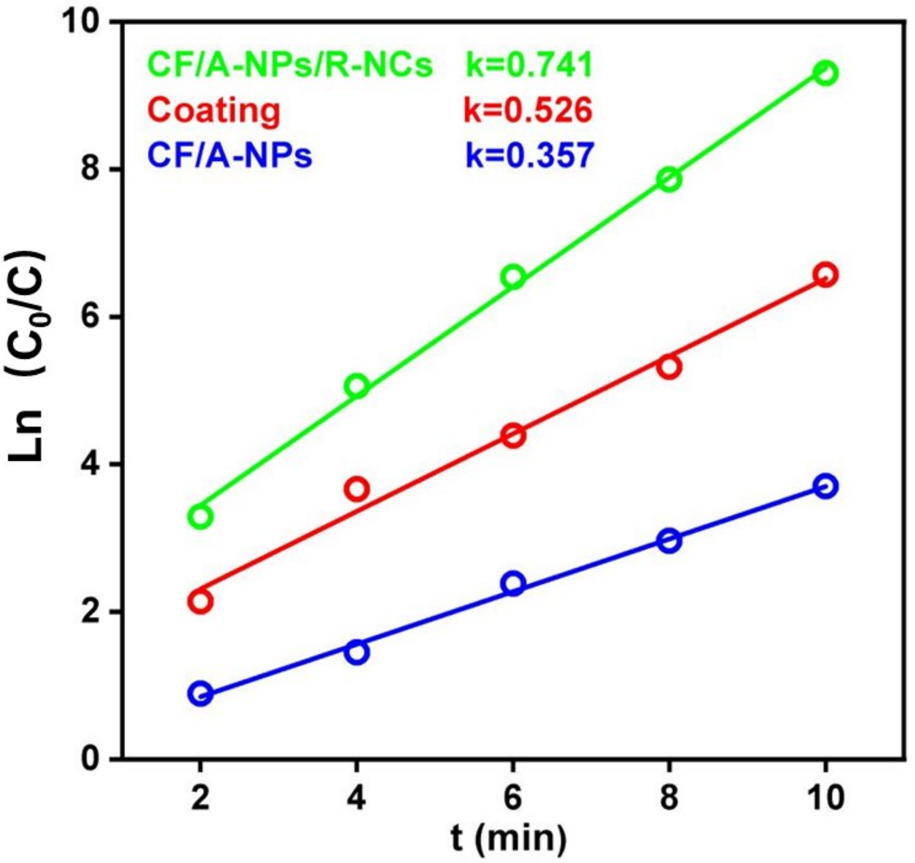

Figure S2. First order kinetic fitting results of photocatalytic uranium removal for three materials.

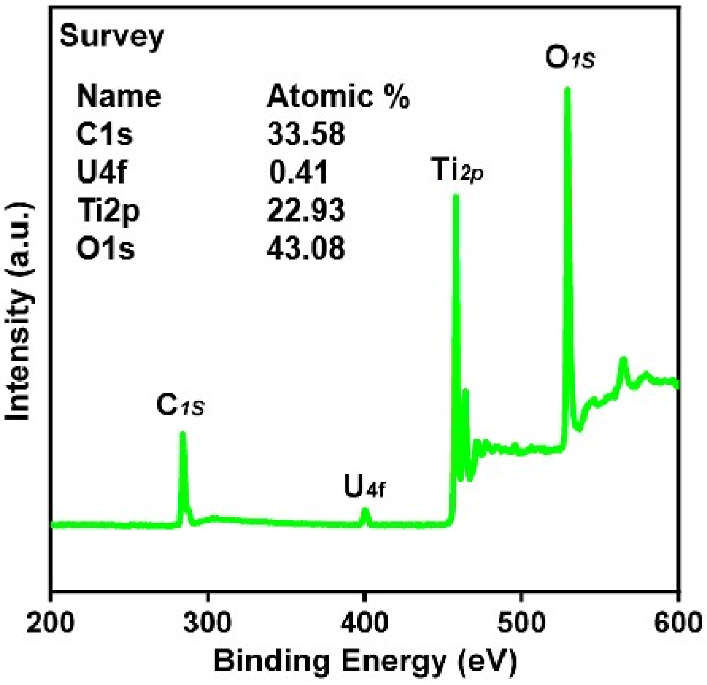

Figure S3. XPS spectrum of the sample surface after photocatalytic uranium extraction.

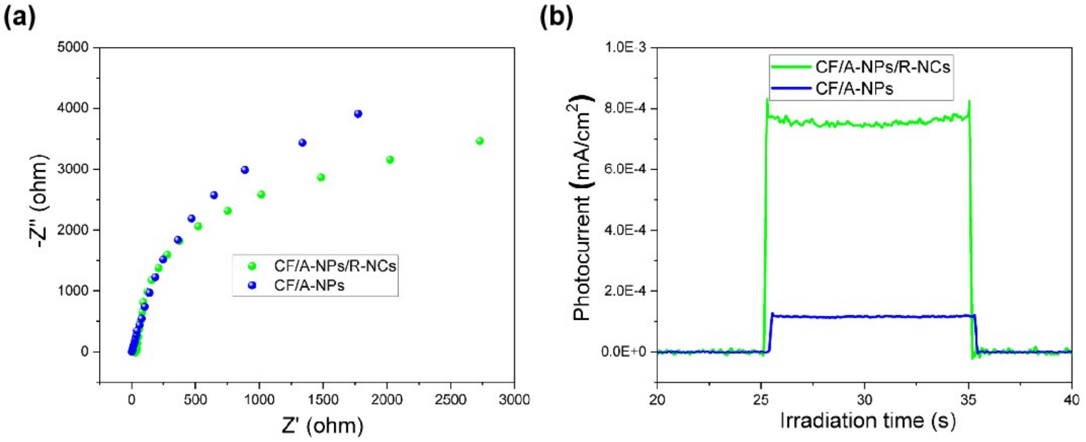

Figure S4. (a) Impedance curve and (b) photocurrent curve of the samples.

References

1. Xu, Y.; Zhang, H.; Liu, Q.; Liu, J.; Chen, R.; Yu, J.; Zhu, J.; Li, R.; Wang, J. Surface Hybridization of π-Conjugate Structure Cyclized Polyacrylonitrile and Radial Microsphere Shaped TiO_2 for Reducing U(VI) to U(IV). *J. Hazard. Mater.* **2021**, *416*, 125812. https://doi.org/10.1016/j.jhazmat.2021.125812.
2. Li, M.; Wang, R.; Liu, T.; Chen, Q.; Li, N.; Zhou, L.; Yu, K.; Liu, H.; Gong, X.; He, R.; et al. Integrating Surface Functional Modification and Energy-Level Adapted Coupling of Photocatalyst with Ultrafast Carrier Separation for Uranium Extraction. *Sep. Purif. Technol.* **2023**, *309*, 123121. https://doi.org/10.1016/j.seppur.2023.123121.
3. Wang, C.; Jiao, H.; Wu, Y.; Na, P. Amidoxime-Functionalized TiO_2 Nanotube Arrays Plate Photocatalyst for Efficient Extracting and Recovering Uranium from Salt Lakes: Bench-Scale Experiments and Theoretical Calculation. *Sep. Purif. Technol.* **2024**, *339*, 126556. https://doi.org/10.1016/j.seppur.2024.126556.
4. Ma, X.; Meihaus, K.R.; Yang, Y.; Zheng, Y.; Cui, F.; Li, J.; Zhao, Y.; Jiang, B.; Yuan, Y.; Long, J.R.; et al. Photocatalytic Extraction of Uranium from Seawater Using Covalent Organic Framework Nanowires. *J. Am. Chem. Soc.* **2024**, *146*, 23566–23573. https://doi.org/10.1021/jacs.4c07699.
5. Pan, J.; Xiao, B.; Zhu, W.; Yang, Y.; Huang, H.; Lian, Z.; Zhang, T.; Qiu, F.; Xue, S.; Pang, H. Photocatalytic Uranium Extraction Boosted by Dual Effective Active Sites of Porphyrin Metal-Organic Frameworks. *Nano Res.* **2024**, *17*, 6713–6720. https://doi.org/10.1007/s12274-024-6654-x.
6. Xu, M.; Yu, F.; Liu, Y.; Li, W.; Li, C.; Song, F.; Wu, G.; Xu, Z.; Qiu, J. Integrating Uranyl-Affinity "Hooks" into Conjugated Polymers Achieving Giant Built-in Electric Field for Boosting Photocatalytic Uranium Extraction from Seawater. *Macromolecules* **2024**, *57*, 5679–5690. https://doi.org/10.1021/acs.macromol.4c00717.
